# Genetic architecture of terpene chemistry and growth traits and the impact of inbreeding on these traits in western redcedar (*Thuja plicata*)

**DOI:** 10.1111/eva.13526

**Published:** 2023-01-12

**Authors:** Tal J. Shalev, Omnia Gamal El‐Dien, Macaire M. S. Yuen, Lise van der Merwe, Matias Kirst, Alvin D. Yanchuk, Carol Ritland, John H. Russell, Joerg Bohlmann

**Affiliations:** ^1^ Michael Smith Laboratories University of British Columbia Vancouver British Columbia Canada; ^2^ Pharmacognosy Department, Faculty of Pharmacy Alexandria University Alexandria Egypt; ^3^ British Columbia Ministry of Forests Victoria British Columbia Canada; ^4^ School of Forest, Fisheries and Geomatic Sciences University of Florida Gainesville Florida USA

**Keywords:** genomics, growth, inbreeding, quantitative genetics, terpenes, western redcedar

## Abstract

Western redcedar (WRC; *Thuja plicata*) is a conifer of the Pacific Northwest of North America prized for its durable and rot‐resistant wood. WRC has naturally low outcrossing rates and readily self‐fertilizes in nature. Challenges faced in WRC breeding and propagation involve selecting trees for accelerated growth while also ensuring enhanced heartwood rot resistance and resistance to ungulate browsing, as well as mitigating potential effects of inbreeding depression. Terpenes, a large and diverse class of specialized metabolites, confer both rot and browse resistance in the wood and foliage of WRC, respectively. Using a Bayesian modelling approach, we isolated single nucleotide polymorphism (SNP) markers estimated to be associated with three different foliar terpene traits and four different heartwood terpene traits, as well as two growth traits. We found that all traits were complex, being associated with between 1700 and 3600 SNPs linked with putatively causal loci, with significant polygenic components. Growth traits tended to have a larger polygenic component while terpene traits had larger major gene components; SNPs with small or polygenic effect were spread across the genome, while larger‐effect SNPs tended to be localized to specific linkage groups. To determine whether there was inbreeding depression for terpene chemistry or growth traits, we used mixed linear models for a genomic selection training population to estimate the effect of the inbreeding coefficient *F* on foliar terpenes, heartwood terpenes and several growth and dendrochronological traits. We did not find significant inbreeding depression for any assessed trait. We further assessed inbreeding depression across four generations of complete selfing and found that not only was inbreeding depression not significant but that selection for height growth was the only significant predictor for growth during selfing, suggesting that inbreeding depression due to selfing during operational breeding can be mitigated by increased selection intensity.

## 1 INTRODUCTION

Western redcedar (*Thuja plicata*; WRC) is an ecologically, economically and culturally important conifer of the Cupressaceae family. WRC is endemic to North America's Pacific Northwest and is held in high regard by coastal First Nations and Native Americans, for whom WRC has served for millennia as a keystone societal resource. WRC wood has been used in the construction of buildings, watercrafts, totem poles and ceremonial masks, among other important practical and spiritual uses (Hebda & Mathewes, 1984). It has also found extensive modern use in exposed building applications due to its durable, lightweight and naturally rot‐resistant wood (Gonzalez, 2004).

Although it currently ranges from Northern California to Southern Alaska, WRC is thought to have expanded its range since the last glaciation from a single refugium south of its current distribution (O'Connell et al., 2008). This expansion may have been facilitated by WRC's unique mating system among conifers: WRC readily propagates via self‐fertilization and appears to be largely unaffected by inbreeding depression for fitness traits (Russell et al., 2003; Russell & Ferguson, 2008; Wang & Russell, 2006). Despite low genetic diversity and small effective population size in natural populations, WRC is responsive to natural and artificial selection, and thrives in a wide range of environmental and climatic conditions (Antos et al., 2016; Grime, 1977), likely due to strong genome‐wide balancing selection (Shalev et al., 2022).

Western redcedar timber has, in the past, been largely harvested from primary old‐growth forests in Canada. To improve sustainability of harvesting practices, there has been momentum to adopt planting and harvesting of second‐growth WRC. However, WRC seedlings grown in plantations are vulnerable to heavy ungulate browsing, greatly reducing survival; furthermore, second‐growth WRC have been found to be susceptible to a host of fungal pathogens, such as those responsible for cedar leaf blight and heartwood rot (Gonzalez, 2004). Thus, Canada's WRC tree improvement programme is currently focused on breeding trees for improved growth, heartwood durability and resistance to disease and herbivores (Russell & Yanchuk, 2012). Previous studies have shown that monoterpenes (specialized metabolite compounds containing 10 carbon atoms) deposited in oil glands in WRC foliage deter browsing of ungulates (Vourc'h, De Garine‐Wichatitsky, et al., 2002; Vourc'h, Russell, et al., 2002). Similarly, monoterpenoid tropolones called thujaplicins present in the heartwood of WRC have been implicated in the tree's natural defence against fungal pathogens (Barton & Macdonald, 1971; Van der Kamp, 1986).

Terpenes are a class of compounds derived from the five‐carbon building blocks isopentenyl diphosphate (IPP) and dimethylallyl diphosphate (DMAPP). Terpenes constitute a large group of metabolites in plants, with over 40,000 structures involved in both general and specialized metabolism (Bohlmann & Keeling, 2008). They are particularly abundant and diverse in the chemical defences of various conifers (Celedon & Bohlmann, 2019). The first two steps in the biosynthetic pathway of α‐thujone, the most abundant foliar monoterpene in WRC, have been identified, but the genes involved in the final steps of the pathway have yet to be identified (Foster et al., 2013; Gesell et al., 2015). Compared with foliar monoterpenes, much less is known about the pathways of thujaplicin biosynthesis. Only the pathway of β‐thujaplicin has been studied in some depth (Ko et al., 2015; Liu & Yamauchi, 2006; Tu et al., 2016; Zhang et al., 2019), and the genes involved in the majority of the pathway, including the formation of the seven‐membered tropolone ring, remain unknown.

Developing our knowledge of the genetic architecture (i.e. the underlying genetic basis of a trait and the genetic properties which lead to variation in a trait) and molecular mechanisms controlling these growth and chemistry traits in WRC will be essential for understanding how WRC grows and adapts to pest and pathogen attacks, and for improving the growth and survival prospects of trees in the wild and during operational forestry. It is also unknown which genomic loci may have an impact on inbreeding depression, or lack thereof, in growth or chemistry traits in WRC. Recently, large‐scale genotyping of WRC has been carried out for the implementation of genomic selection for growth and chemistry traits in WRC, with the goal of expediting breeding cycles for these traits (Gamal El‐Dien et al., 2022). Given WRC's unique mating system and low genetic diversity (Shalev et al., 2022), a better understanding of the areas of the genome controlling traits of interest would be beneficial in optimizing the process of genomic selection, and for learning and understanding more about this important tree species and the genetic control of chemistry and growth traits in general.

The objectives of this study were to use recently developed genomic resources to identify genomic regions associated with growth and monoterpene chemistry traits in WRC, and determine whether there is inbreeding depression for these traits and whether strong selection could override the effect of inbreeding. We hypothesize that, given the quantitative nature of growth and chemistry traits, these traits will most likely be controlled by many genomic loci, and that given WRC's selfing capabilities, inbreeding depression will likely be weak, potentially allowing for selection and breeding for the traits examined here even in inbred trees.

## 2 MATERIALS AND METHODS

### 2.1 Training population

The training population (TP) for genomic selection was comprised of 1520 progeny trees from 26 half‐sib families in a polycross mating design growing at three different British Columbia Ministry of Forests (BCMoF) progeny test sites in BC, Canada. A total of 520, 520 and 480 trees were sampled from sites containing large progeny trials at Jordan River, Powell River and Port McNeill respectively (Gamal El‐Dien et al., 2022). Trees were sampled in an incomplete block design, with trees from the same family at different sites considered as ‘replicates’, and each ‘block’ containing a single ‘replicate’ from each family. On average, 20 trees per polycross family were selected from each site. For further details, see Gamal El‐Dien et al. (2022).

### 2.2 Selfing lines

Breeding and propagation of selfing lines (SLs) were carried out between 1995 and 2007 at the Cowichan Lake Research Station (CLRS) at Lake Mesachie, British Columbia (BC), Canada. Fifteen pairs (30 individuals) of unrelated grafted parent trees taken originally from various locations across Coastal BC and Vancouver Island (Shalev et al., 2022) and growing at CLRS were crossed to generate 15 full‐sib (FS) families. This was done to ensure that all lines would initialize with an inbreeding coefficient of *F* = 0. A total population of 60 seedlings was generated for selfing in the accelerated breeding cycle as described by Russell and Ferguson (2008). Briefly, in the 1st year, the seed was germinated in the greenhouse, grown for 5 months before repotting and induction of flowering with gibberellic acid (GA_3_), and then moved outside for chilling. In the 2nd year, trees were moved back into the greenhouse and were completely encased in pollination bags to allow for selfing, and cones were then collected to begin the process again, allowing for one generation every 2 years. Seedlings from each FS family were planted in three replicates in a greenhouse.

Shoot heights were measured after 4 months, and the two seedlings with the top growth per family were selected based on standard normal deviates; this selection continued at each generation to form the ‘selected’ lines. A further two seedlings per FS family were selected at random at each generation; these formed the ‘random’ lines. Three trees from each line were chosen at each generation (S1–S5) and brought through the entire selfing process to mitigate the likelihood of line extinction. Ten of the 60 lines went extinct during the process. Foliar tissue was collected for DNA extraction and foliar chemistry analysis from FS – S4 trees between 4 and 15 years of age growing in outdoor plots at CLRS; and 1‐year‐old S5 seedlings growing in a greenhouse at CLRS, for a total of *n* = 191 trees in *n* = 41 SLs. For a detailed sampling schematic, see Figure S1.

### 2.3 DNA extraction

DNA was isolated as described by Shalev et al. (2022). Briefly, a modified protocol of Xin and Chen (2012) was followed, but with the addition of 10% polyvinylpyrrolidone to the CTAB extraction buffer, all centrifugation steps were performed for 10–12 min at 7,826 *g*, the amount of Qiagen MagAttract solution was increased to 7 μl, 1‐min wait time for binding on the magnetic block was added and 115 μl TE buffer was used for DNA dilution. The concentration and quality of DNA were verified with a Nanodrop 2000c (Fisher Scientific), Quantiflor (Promega Corporate) and 0.8% agarose gel to cross‐check for the quantity and quality of the DNA.

### 2.4 SNP genotyping and filtering

Targeted sequencing‐based genotyping was done by Capture‐Seq methodology at Rapid Genomics, as described by Shalev et al. (2022). Briefly, we designed probes to target regions of putative high variability, and specifically the following: previously identified differentially expressed regions from cold‐tolerance, deer browse, wood durability, leaf blight and growth trials, database matches for functionally characterized conifer genes, and whole‐transcriptome and genome data (NCBI Umbrella BioProject PRJNA704616) (Shalev et al., 2018, 2022). From a total of 57,000 probes, we selected 20,858 probes with at least 17 SNPs per probe, 16,189 (77%) in genic regions and 4669 (23%) in intergenic regions, for SNP calling. SNPs were then called for a total of 4832 trees, comprising TP and SL, as well as the target population and parent trees for genomic selection (Shalev et al., 2022).

We filtered SNPs using vcftools v0.1.17 (Danecek et al., 2011) with the following flags: ‐‐max‐missing 0.95; ‐‐minQ 30; ‐‐min‐meanDP 15; ‐‐max‐meanDP 60 and ‐‐maf 0.01. SNPs with an allele balance (the ratio of reference allele reads to overall reads in heterozygous individuals) greater than 0.25 and lower than 0.75, or lower than 0.01 or greater than 0.99, were retained using vcffilter in vcflib (Garrison et al., 2022) to eliminate incorrectly called heterozygotes. Samples with more than 40% missing data were removed. A stringent maximum mean depth of 60× was chosen to reduce the likelihood of erroneous calls due to sequence paralogy. SNPs were then filtered for excess heterozygosity at a *p*‐value cut‐off of 0.001.

For SLs, further genotype correction was carried out as described in Shalev et al. (2022), using SNPs filtered for all above criteria except minor allele frequency (MAF). Briefly, 20 lines with available SNP data from multiple consecutive generations were corrected if a heterozygous call in a later generation followed consecutive homozygous calls in previous generations, or if a homozygous call for one genotype followed consecutive homozygous calls for the other genotype. SNPs that could not be corrected following these criteria were removed. For seven SLs in which only the S3 generation had not been sequenced, we imputed the genotypes for the S3 generation at each locus for SNPs where no other genotype was possible, and marked the rest as missing.

SNPs were converted to genotype dosage matrix using the ‐‐012 flag in vcftools. A genomic realized relationship matrix (GRRM) was generated for both the TP and the selfing lines using the ‘A.mat’ function from rrBLUP v4.6.1 (Endelman, 2011) in R v4.1.2 (R Core Team, 2021). SNPs were annotated using the Ensembl Variant Effect Predictor r103 (McLaren et al., 2016) and the draft genome annotation (Shalev et al., 2022).

### 2.5 Phenotyping

In our analysis of WRC traits in the TP, we focused on the most abundant foliar monoterpenes (α‐thujone, β‐thujone and sabinene), wood thujaplicins (α‐, β‐, γ‐thujaplicin and β‐thujaplicinol) and height and diameter at breast height at 15 years of age (Dataset S1). For the SLs, we focused on foliar monoterpenes and height at 4 months of age.

#### 2.5.1 Growth

Height and diameter at breast height (DBH, ~1.3 m) were measured for trees in the TP at approximately 15 years of age in 2014. Height was measured for 4‐month‐old seedlings of all SLs up to S4 between 1997 and 2005.

#### 2.5.2 Foliar chemistry

Foliar chemistry was analysed from foliar tissue sampled from TP trees at 15 years of age and SL trees between 1 and 15 years of age at Glynn Road Analytical Chemistry Lab, as described in Gamal El‐Dien et al. (2022). Briefly, sample extraction and analysis followed the method of Kimball et al. (2005) with modifications. Frozen samples were ground using liquid nitrogen and a mortar and pestle. Approximately 250 mg of wet weight sample was extracted into 4 ml of methanol containing pentadecane as an internal standard. An additional subsample was measured for moisture factor to provide a 70°C oven dry weight measurement. Samples were extracted for 48 h and analysed on a Clarus 580 Gas Chromatography (GC) unit (PerkinElmer) with a 30 m ZB‐5MSi GC column using flame ionization detection (FID). Peaks were identified by comparing retention times to reference standards, and mass spectrometry (MS) was used for further confirmation when necessary.

#### 2.5.3 Wood chemistry

For wood chemistry, increment cores were sampled from TP trees at 18 years of age in the fall of 2017. Cores were taken at breast height (1.3 m) and were oriented to intercept the pith of the stem to ensure all heartwood material was captured in the sample while capturing the entire diameter of the tree. One‐half (one radius) of each core was retained for dendrochronological analysis. The other half was ground for 5–10 s in an electric grinder. The grinder was swept after each sample and cleaned with methanol after every 10th sample. Ground samples were conditioned at 40°C for 48 h before extraction to ensure consistency between samples, as the small sample weights did not allow for individual moisture contents.

Extraction and analysis of wood metabolites were carried out at FPInnovations using the approach of Daniels and Russell (2007) with some modifications, as described by Gamal El‐Dien et al. (2022). Briefly, gradient elution from 10% acetonitrile to 65% acetonitrile in 44 min at a flow rate of 1.4 ml/min was used for all analyses. Samples were extracted in 1–2 ml of ethanol using crotonic acid para‐bromophenacyl as an internal standard. Samples were sonicated in an ultrasonic bath for 2 h and 0.5 ml of sample extract was placed in a filter vial for analysis by reverse‐phase liquid chromatography using an Agilent 1260 HPLC (Agilent Technologies). Reference standards were used for the identification of terpene compounds.

#### 2.5.4 Dendrochronology

For dendrochronology, each core was scanned into the program Coordinate Recorder (Cybis Elektronik & Data AB). Each image was assessed to (1) identify ring boundaries and detect false rings (e.g. intra‐annual bands of latewood cells); and (2) assign calendar years to each ring, assuming the last ring with complete latewood was 2016 for all samples. A constant threshold was applied to automatically detect the earlywood–latewood boundary within each ring. Three time series of data representing total ring, earlywood and latewood widths were recorded for each tree.

The calendar year of the pith or inner ring was recorded. The number of years from this year to sampling date was designated as ‘pith age’. The majority of cores intercepted the pith and included the bark. Ring counts represented age of the trees (at coring height) and ring widths were summed to represent growth over their lifespan. For cores that included arcing rings adjacent to the pith, we calculated correction factors for the missed portion of the radius and number of rings (Duncan, 1989):

(1) $$\text{Missed portion of the radius}=\frac{{\text{Length}}^{2}}{8\times \text{Height}}+\left(\text{Height}+2\right)$$

where the length and height (mm) of the arcing latewood of the inner ring were measured with digital callipers; and

(2) $$\text{Number of missed rings}=\frac{\text{Missed portion of the radius}}{\text{Average ring width}}$$

where the width of the three rings closest to the pith was averaged.

Using these corrections, the radius from the pith to the latewood of the 2016 ring was calculated by summing the measured ring widths and adding the estimated missed portion of the radius. Total number of rings was calculated as the number of measured rings plus the estimated number of missing rings. For cores missing the bark, there is no standard method to estimate the number of potentially missing sapwood rings, so no correction was applied to address this potential source of error. Their ring counts and summed ring widths provide minimum estimates of age and growth. The number of sapwood rings per sample was counted and ring widths were summed. The number of heartwood rings was calculated as total number of rings minus number of sapwood rings. Heartwood width was calculated as total radius minus sapwood width.

#### 2.5.5 Estimation of narrow‐sense heritability (*h* ^2^) and genetic correlations

We estimated narrow sense heritability (*h* ^2^) for our traits in the TP from SNP data using univariate mixed linear models (genomic best linear unbiased predictors; GBLUP) and from our corrected pedigree data (ABLUP) in ASReml‐R v4.1.0.149 (Butler et al., 2017):

(3) y=Xβ+Za+ε

where y is an *n*‐dimensional vector of phenotypes, β is a *p*‐dimensional vector of fixed effects, X is an *n* × *p* design matrix relating phenotypes to fixed effects, a is an *n*‐dimensional vector of random effects including the inverse GRRM from SNP data or the average relationship matric from pedigree, Z is an *n* × *n* identity matrix relating phenotypes to unique individual effects and ε is an *n*‐dimensional vector of residual errors with variance $I\sigma_{e}^{2}$. Mixed linear models were used to account for incomplete block study design and make inferences on genetic parameters for the entire WRC population from families in the TP.

Restricted effects maximum‐likelihood (REML) ratio test was implemented using the asremlPlus v4.2‐18 package in R (https://github.com/briencj/asremlPlus) to test and compare included random effects. The models used for each trait can be found in Table S1. For all models, a heterogeneous variance structure (dsum option in ASReml‐R) was used to split the residual variance by site.

We calculated *h* ^2^ as $\sigma_{g}^{2}/\left(\sigma_{g}^{2}+\sigma_{e}^{2}\right)$, where $\sigma_{g}^{2}$ is the additive genetic variance from the model and $\sigma_{e}^{2}$ is the residual variance. In cases where a heterogeneous residual variance structure was used, $\sigma_{e}^{2}$ was calculated by taking the mean of the residual variance components. Standard errors were calculated using the ‘delta’ approach of Venables and Ripley (2000) as implemented in the ‘vpredict’ function in ASReml‐R.

Genetic and phenotypic correlations were estimated using bivariate mixed linear models for each pair of traits in ASReml‐R. An unstructured variance model was used for the inverse RRM. Genetic correlations were calculated from both genomic and pedigree data as $r_{g}={\mathrm{cov}}_{g}/\sqrt{V_{g1}V_{g2}}$, where $V_{g1}$ and $V_{g2}$ are the additive genetic variances of each trait in the model and ${\mathrm{cov}}_{g}$ is the additive genetic covariance of these traits. Phenotypic correlations were calculated as $r_{p}=\left({\mathrm{cov}}_{g}+{\mathrm{cov}}_{e}\right)/\sqrt{\left(V_{g1}+V_{e1}\right)\left(V_{g2}V_{e2}\right)}$, where $V_{e1}$ and $V_{e2}$ are the residual variances of each trait in the model and ${\mathrm{cov}}_{e}$ is the residual covariance of these traits.

#### 2.5.6 Adjusted phenotypes for BayesR

To simplify our model for use in BayesR, we used an adjusted phenotype for each trait in the TP to train the Bayesian model. This adjusted phenotype was obtained using ASReml‐R by extracting the residual of model (3), as described above (Dataset S2). In this case, the random effect for additive genetic component was excluded from the model. Fixed effects were tested for significance using a Wald test.

#### 2.5.7 Genetic architecture of chemistry and growth traits

BayesR (Erbe et al., 2012; Moser et al., 2015) was used to identify SNPs linked with putatively causal loci (hereafter, trait‐associated SNPs) and estimate genetic architecture of our traits of interest in the TP. BayesR allows us to estimate the variance explained by all SNPs and thus the effects of individual SNPs by fitting all SNPs in the model simultaneously (Moser et al., 2015). We implemented BayesR assuming the default prior mixture of four normal distributions with *N*(0, 0 × $\sigma_{g}^{2}$) (no additive genetic effect), *N*(0, 10^−4^ × $\sigma_{g}^{2}$) (polygenic effect), *N*(0, 10^−3^ × $\sigma_{g}^{2}$) (small effect) and *N*(0, 10^−2^ × $\sigma_{g}^{2}$) (large effect), respectively, where $\sigma_{g}^{2}$ is the additive genetic variance.

Five independent chains were run for each trait to ensure model convergence and to obtain averaged posterior estimates, using the following settings in BayesR: ‐numit 300000; ‐burnin 100000; ‐thin 10 and ‐permute. The convergence of the model was inspected using trace plots for *h* ^2^ and each posterior distribution, and the Gelman–Rubin convergence diagnostic (Brooks & Gelman, 1998; Gelman & Rubin, 1992). SNP effect size (ES) was calculated using the equation

(4) $$\mathrm{ES}=2\beta^{2}f\left(1-f\right)$$

where β is the regression effect of the model and f is the frequency of the minor allele (Park et al., 2010). ES and mixture distributions were averaged across all five chains and SNPs were assigned to each mixture distribution based on the estimated number of SNPs for each distribution from the model and SNP ES.

After ranking and extracting all SNPs with some effect for each trait, we then generated set plots using UpSetR v1.4.0 in R (Conway et al., 2017) to isolate and further annotate and explore SNPs that overlapped between different traits. In particular, we looked at (1) shared SNPs between traits with significant genetic correlations; (2) SNPs unique to any single category of traits (foliar chemistry, wood chemistry or growth) and (3) SNPs shared between trait categories.

To estimate localization of SNPs across the genome, we used putative linkage groups (LGs), generated previously for the WRC genome (Shalev et al., 2022) by aligning all genomic scaffolds containing SNPs to the *Sequoiadendron giganteum* (giant sequoia) draft genome (Scott et al., 2020) using BLAST+ v2.10.0 (Altschul et al., 1990; Camacho et al., 2009). Briefly, scaffolds were assigned to their most likely LG based on bitscore. We then used the nucmer command from MUMmer v4 (Marçais et al., 2018) to determine the most likely alignment region for each scaffold in each LG.

### 2.6 Inbreeding depression of growth and chemistry traits

#### 2.6.1 Inbreeding depression in the training population

Another goal of our study was to determine whether WRC shows evidence of inbreeding depression for growth and chemistry traits. We first calculated inbreeding coefficients in PLINK v1.9 (Chang et al., 2015) using the ‐‐ibc flag to obtain a measure for inbreeding based on the correlation between uniting gametes (*F*_UNI_). This metric, designated Fhat3 in PLINK, is defined by Yang et al. (2011) for each ith SNP and each jth individual as:

(5) $$F_{\mathrm{UNI}}=\frac{x_{\mathrm{ij}}^{2}-\left(1+2p_{i}\right)x_{\mathrm{ij}}+2p_{i}^{2}}{2p_{i}\left(1-p_{i}\right)}$$

where x is the number of copies of the reference allele and p is the population‐wide allele frequency at that locus. SNPs were filtered for linkage disequilibrium (LD) using PLINK (flags: ‐‐indep‐pairwise 2168 kb 1 0.1) based on previous estimates of LD in WRC (Shalev et al., 2022) prior to estimation of inbreeding coefficients in the TP and SLs.

To examine whether there was significant inbreeding depression for growth traits and chemistry traits, we used mixed linear models for our TP, following the same approach used to obtain adjusted phenotypes, and added *F*_UNI_ as a covariate in the model (Table S1). For this analysis, we examined total foliar monoterpenes, total wood thujaplicins, height, DBH, heartwood radius, sapwood radius and the ratio of sapwood to heartwood. A Wald test was then used to test for significance of *F*_UNI_.

#### 2.6.2 Analysis of growth and chemistry traits during selfing

Using SLs, we further aimed to explore the effect of selection for height during extreme inbreeding, and identify which SNPs associated with growth and chemistry traits identified in this study may be under selection during selfing.

Due to limitations in data availability, we only had access to breeding values (BVs) for height in our SLs. BVs for height were predicted for each generation in each line as described in Russell and Ferguson (2008). Briefly, BVs were predicted by ABLUP using a mixed linear model (3), where y is an *n*‐dimensional vector of raw phenotypes, X is an *n* × *p* design matrix for fixed effects, β is an *n*‐dimensional vector of fixed effects, Z is an *n* × *p* design matrix for random effects, a is an *n*‐dimensional vector of random effects including the inverse relationship matrix from pedigree and ε is an *n*‐dimensional vector of residuals. Specifically, the following model was used:

(6) $$y_{\text{ijkl}\left(m\right)n}=u+g_{i}+g_{i}\left(r_{j}\right)+t_{k}+g_{i}t_{k}+f_{l}+g_{i}f_{l}+f_{l}\left(l_{m}\right)+g_{i}f_{l}\left(l_{m}\right)+e_{\text{ijkl}\left(m\right)n}$$

where u is the population mean; $g_{i}$, $g_{i}\left(r_{j}\right)$, $t_{k}$ and $g_{i}t_{k}$ are the fixed effects of the ith selfed generation, the jth replication within the ith selfed generation, the kth line type (random or selected) and the interaction between the ith selfed generation and kth line type, respectively; $f_{l}$, $g_{i}f_{l}$, $f_{l}\left(l_{m}\right)$ and $g_{i}f_{l}\left(l_{m}\right)$ are the random effects of the lth family, the interaction between ith selfed generation and lth family, the mth line within the lth family and the interaction between ith selfed generation and mth line within lth family, respectively; and $e_{\text{ijkl}\left(m\right)n}$ is the random error associated with trees within replication.

BVs for foliar monoterpenes were calculated using a mixed linear model (GBLUP) in ASReml‐R:

(7) $$y_{\mathrm{ijk}}=u+g_{i}+l_{j}+a_{k}+e_{\mathrm{ijk}}$$

where u is the population mean; $g_{i}$ and $l_{j}$ are the fixed effects of the ith selfed generation and the jth growing location at CLRS, respectively; $a_{k}$ is the additive genetic effect of the kth tree as estimated from the inverse GRRM from SNP data and $e_{\mathrm{ijk}}$ is the random error associated with trees within each generation and location.

#### 2.6.3 Inbreeding depression in selfing lines

We assessed significance of *F* for height BVs and total foliar monoterpene BVs in the SLs using ordinary least squares (OLS) in the following linear model:

(8) $$y_{i}=u+F_{i}+e_{i}$$

where u is the population mean, $F_{i}$ is the fixed effect of the ith tree's inbreeding coefficient and $e_{i}$ is the random error associated with inbreeding coefficients. Assumptions of independent and identical distribution of error terms were tested in R using residual plots and normality histograms.

#### 2.6.4 Effect of selection for height on height BVs and inbreeding depression

We used OLS to estimate the effect of selection on height BVs in the following linear model:

(9) $$y_{\mathrm{ijk}}=u+F_{i}+t_{j}+F_{i}t_{j}+f_{k}+e_{\mathrm{ijk}}$$

where u is the population mean; $F_{i}$, $t_{j}$, $F_{i}t_{j}$ and $f_{k}$ are the fixed effects of the ith tree's inbreeding coefficient, the jth line type (random or selected), the interaction between the ith inbreeding coefficient and the jth line type, and the kth family, respectively; and $e_{\mathrm{ijk}}$ is the random error associated with these fixed effects.

## 3 RESULTS

### 3.1 SNP genotyping and filtration

Using a targeted genotyping approach, we captured 51,638 polymorphic loci from 20,858 probes. Filtering across all trees yielded 36,262 SNPs (Dataset S3). Among SNPs in annotated regions, 9855 were in intergenic regions and 24,418 were in genic regions. Fourteen TP trees from families external to the recorded pedigree as identified by the corrected pedigree (Gamal El‐Dien et al., 2022) and two trees with 40% or more missing data were excluded, resulting in a final *n* = 1504. We corrected erroneous SNP calls for the SLs using the approach of Shalev et al. (2022), resulting in continuous SLs to S4 (*n* = 28) or S5 (*n* = 11) with a total of *n* = 151 trees with genotyping data.

### 3.2 Genetic architecture of growth and specialized chemistry traits in western redcedar

#### 3.2.1 Foliar terpenes

Foliar chemistry data were obtained for *n* = 1499 trees. The most abundant foliar monoterpene by far in our TP was α‐thujone, with a mean (SD) concentration of 23,249 (7538) μg/g DW, followed by sabinene with a mean concentration of 5718 (2314) and finally, β‐thujone with a mean of 4732 (1679). There was substantial variation in foliar terpene concentration between trees, with α‐thujone concentrations ranging from 6256 to 57,408, sabinene from 559 to 22,903 and β‐thujone from 1468 to 13,445 μg/g DW (Figure S2A). The sampling site had a significant effect on all foliar terpenes (*p* < 0.01, Wald test).

#### 3.2.2 Wood thujaplicins

Wood chemistry data were obtained for *n* = 1492 trees. Of the four thujaplicins analysed in this study, α‐ and γ‐thujaplicin were the most abundant, with mean (SD) concentrations of 396.5 (275.6) and 361.1 (340.8) μg/g conditioned wood (CW), respectively, followed by β‐thujaplicin with 210.2 (225.6) and β‐thujaplicinol with 67.5 (66.8) μg/g CW. The distribution of these phenotypes was right‐skewed, with more trees exhibiting lower‐than‐average concentrations (Figure S2B). For all wood thujaplicins, sampling site had a significant effect (*p* < 0.001, Wald test). Pith age had a significant effect on α‐ and γ‐thujaplicin and β‐thujaplicinol (*p* < 10^−5^, Wald test). The sapwood‐to‐heartwood ratio had a significant effect on α‐thujaplicin and β‐thujaplicinol (*p* < 0.005, Wald test).

#### 3.2.3 Growth

Both height and DBH were significantly greater overall at the Powell River site, with a mean height (SD) of 1084.5 (156.8) cm and DBH of 143.0 (32.7) mm compared to the means of Jordan River [761.6 (102.0) cm for height and 110.0 (24.5) mm for DBH (*p* = 0, one‐tailed *t*‐test)] and Port McNeill [683.1 (119.2) cm for height and 98.6 (27.7) mm for DBH (*p* = 0, one‐tailed *t*‐test)] (Figure S3; Table S2). Thus, site had a very significant effect on both height and DBH at 15 years (*p* = 0, Wald test).

### 3.3 Heritabilities

We recorded moderate genomic *h* ^2^ for α‐thujone and sabinene (0.27 each), with a lower *h* ^2^ for β‐thujone (0.16) (Table S3). Pedigree‐based estimates of *h* ^2^ for α‐thujone and sabinene were similar but slightly higher (0.35 and 0.39, respectively), while the estimate for β‐thujone was lower (0.14). For wood thujaplicins, β‐thujaplicin had the highest genomic *h* ^2^ (0.30), although the pedigree‐based estimate was lower (0.21). α‐ and γ‐thujaplicin and β‐thujaplicinol all had moderate‐to‐low genomic *h* ^2^ (0.26, 0.19 and 0.13, respectively), with similar pedigree‐based results. Height and DBH both had low genomic *h* ^2^ (0.14 and 0.10, respectively). In all cases, genomic *h* ^2^ had lower standard error than pedigree estimates.

### 3.4 Genetic and phenotypic correlations

As the foliar terpenes in this study are in the same biosynthetic pathway, we hypothesized that genetic and phenotypic correlations between them would be strong. Genetic and phenotypic correlations were similar and high between α‐ and β‐thujone (0.65 and 0.60, respectively) (Table S4). Genetic correlations were moderate between both α‐thujone and sabinene (0.35) and β‐thujone and sabinene (0.30); however, the phenotypic correlation between α‐thujone and sabinene was high (0.66).

Due to the structural similarity of the wood thujaplicins, we expected to observe genetic and phenotypic correlations between them. We recorded moderate‐to‐high genetic and phenotypic correlations between the different wood thujaplicins (Table S4). Overall, β‐thujaplicin had moderately low genetic correlations with γ‐thujaplicin and β‐thujaplicinol (0.22 and 0.20, respectively), but a moderately high genetic correlation with α‐thujaplicin (0.46). Interestingly, γ‐thujaplicin and β‐thujaplicinol had the highest genetic correlation of all traits studied, and a very high phenotypic correlation as well (0.76 each). We observed the greatest discrepancy between genomic and pedigree‐based estimates for wood thujaplicins, with some estimates differing by as much as 0.15 between methods. Height and DBH were strongly genetically and phenotypically correlated (0.75 and 0.85, respectively).

Between groups of traits, the largest significant genetic correlation was observed between β‐thujone and DBH (0.39). There were very small or no significant genetic correlations between wood thujaplicins and other traits, with the exception of sabinene and α‐thujaplicin (0.31). There was a moderate but significant negative genetic correlation between β‐thujaplicinol and α‐thujone (−0.20); there were also negative phenotypic correlations between almost all wood thujaplicins and growth traits (Table S4). Standard errors of genomic estimates were much lower than pedigree estimates, which is expected.

### 3.5 Analysis of genetic architecture reveals genes involved in terpenoid biosynthesis and growth

Using BayesR, we identified trait‐associated SNPs for each of the traits in this study (Table 1). There was a strong negative rank correlation (−0.93) between the number of trait‐associated SNPs and total variance explained by large‐effect SNPs (10^−2^ × $\sigma_{g}^{2}$), suggesting that traits with a greater degree of control by major genes have less loci overall associated with them. There was a strong correlation (0.99) between number of trait‐associated SNPs and number of genes associated with each trait; indeed, only 28% of annotated trait‐associated SNPs were in intergenic regions, indicating that an increase in number of trait‐associated SNPs also entails an increase in the number of genes controlling a trait.

**TABLE 1:** Trait‐associated SNPs and effect sizes for each trait estimated using the approach of BayesR (Moser et al., 2015)

Trait	Trait‐associated SNPs	Large‐effect SNPs (10^−2^ × $\sigma_{g}^{2}$)	Small‐effect SNPs (10^−3^ × $\sigma_{g}^{2}$)	Polygenic SNPs (10^−4^ × $\sigma_{g}^{2}$)	No effect SNPs (0 × $\sigma_{g}^{2}$)	Annotated genes
α‐Thujone	2206	60	249	1897	34,056	929
β‐Thujone	2704	54	270	2380	33,558	1085
Sabinene	3602	28	445	3129	32,660	1417
α‐Thujaplicin	2508	55	279	2174	33,754	1017
β‐Thujaplicin	2559	52	319	2188	33,703	1018
γ‐Thujaplicin	1787	67	169	1551	34,475	757
β‐Thujaplicinol	2026	70	191	1765	34,236	930
Height	3016	47	280	2689	33,246	1133
DBH	3505	46	303	3155	32,758	1344

*Note*: Of the 36,262 SNPs used in the study, 14,840 were associated with traits. Of these, 10,178 were in genic regions and 3933 were in intergenic regions, while 729 had no annotation.

**FIGURE 1:**
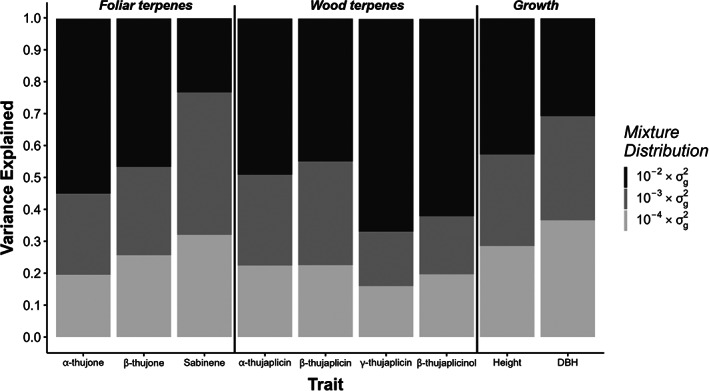
Variance explained by each of three mixture distributions ($\sigma_{g}^{2}$ × 10^−2^ = large effect; $\sigma_{g}^{2}$ × 10^−3^ = small effect and $\sigma_{g}^{2}$ × 10^−4^ = polygenic effect) for each trait as determined by the approach of BayesR (Moser et al., 2015).

Height and DBH were expected to be mainly polygenic, given their highly quantitative nature (Boyle et al., 2017; Yang et al., 2010). We found that this was largely true, with 29% and 37% of the variance in height and DBH, respectively, explained by SNPs in genes with a polygenic effect (Figure 1). Monoterpene traits displayed a range of genetic architectures. Most had a larger major gene component, with γ‐thujaplicin and β‐thujaplicinol having a large amount of variance explained by SNPs with large effect (67% and 62%, respectively). Sabinene, on the other hand, had the largest component of small‐effect SNPs (45%), with only 23% of variance explained by large‐effect SNPs.

#### 3.5.1 SNPs and genes associated with terpene chemistry and growth traits

**FIGURE 2:**
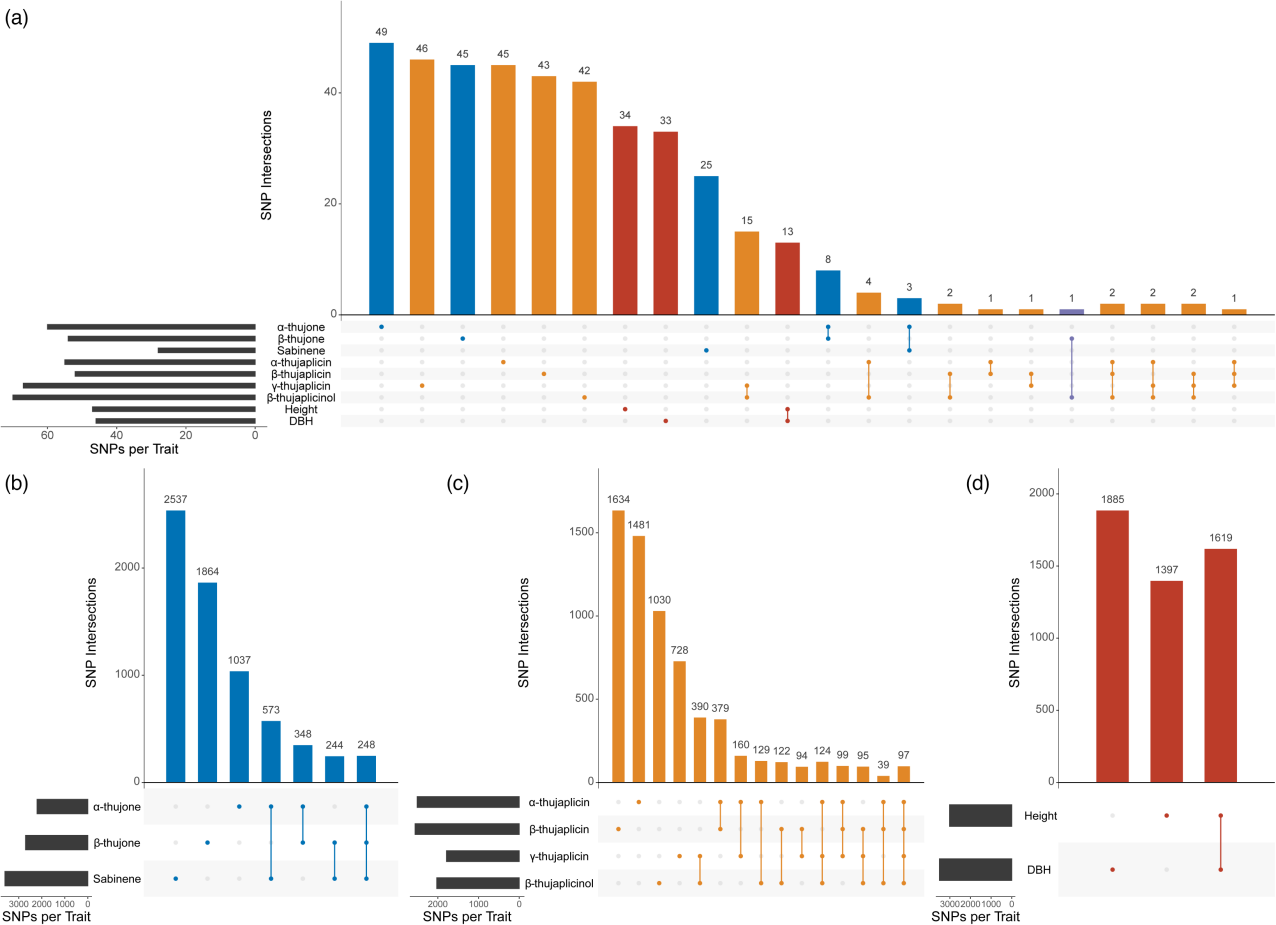
Overlap of trait‐associated SNPs for growth and terpene chemistry traits in WRC. Single dots indicate SNPs unique to each trait. Dots with connecting lines indicate SNPs shared between traits. (a) Overlap of SNPs with large effect between all assessed traits. Blue: foliar terpene traits; Yellow: wood terpene traits; Red: growth traits; Purple: traits of different categories. (b) Overlap of all trait‐associated SNPs for foliar terpene traits. (c) Overlap of all trait‐associated SNPs for wood terpene traits. (d) Overlap of all trait‐associated SNPs for growth traits.

Using our knowledge of the genetic architecture of and genetic correlations between our traits of interest, we further sought to identify the SNPs that are associated with each trait and shared between traits with significant genetic correlations (Figure 2a). We used the current WRC genome annotation (Shalev et al., 2022) to identify which of these SNPs were in genic regions (Table 1).

In particular, to better understand and identify candidates for the final steps of thujone biosynthesis, we isolated genes associated with α‐ and β‐thujone and sabinene, as well as genes with SNPs shared between α‐ and β‐thujone, but not sabinene, which occurs earlier in the biosynthetic pathway, and finally, genes shared by all three traits (Figure 2b). Thujone biosynthesis proceeds via the universal monoterpene precursor geranyl diphosphate (GPP), which is converted into the monoterpene (+)‐sabinene by (+)‐sabinene synthase (Foster et al., 2013), followed by hydroxylation to (+)‐trans‐sabinol by the cytochrome P450 (+)‐sabinene hydroxylase (Gesell et al., 2015). The two proposed remaining steps are oxidation of the (+)‐trans‐sabinol intermediate to (+)‐sabinone, which is subsequently reduced to both the major (α‐thujone) and minor (β‐thujone) products; these reactions are catalysed by a putative alcohol dehydrogenase and aldo/keto reductase respectively (Gesell et al., 2015; Karp & Croteau, 1982). We found one putative alcohol dehydrogenase and two putative aldo/keto reductase genes containing SNPs associated with α‐thujone. Meanwhile, one alcohol dehydrogenase gene was associated with β‐thujone, while no aldo/keto reductase genes were associated with β‐thujone.

For all SNPs associated with each trait, 596 SNPs were shared between α‐ and β‐thujone, of which 348 were not also shared with sabinene (Figure 2b). Of those not shared with sabinene, 256 were in 177 genes. Of SNPs shared between all three foliar terpenes, 173 were in 113 genes (Dataset S1). Interestingly, many more SNPs were shared between α‐thujone and sabinene (821) than between β‐thujone and sabinene (492); this may suggest shared regulatory factors between sabinene and α‐thujone not associated with β‐thujone, providing a possible explanation for why α‐thujone is produced in much greater abundance than β‐thujone. For large‐effect SNPs, only eight SNPs were shared between α‐ and β‐thujone, and none were shared between all three foliar terpenes. Of the eight, four were in four different genes, including a putative methyltransferase and glutamine amidotransferase.

For wood thujaplicins, we were interested in SNPs shared between α‐ and β‐thujaplicin and between γ‐thujaplicin and β‐thujaplicinol, as these have significant genetic correlations, and between all four wood thujaplicins (Figure 2c). There were 614 SNPs shared between α‐ and β‐thujaplicin, 428 of which were in 278 genes. γ‐thujaplicin and β‐thujaplicinol, which had the highest genetic correlation of all traits (0.76), shared 706 SNPs, 520 of which were in 353 genes (Table 1). Only 97 SNPs were shared between all four wood thujaplicins; however, 221 were shared between just α‐ and γ‐thujaplicin and β‐thujaplicinol, indicating that β‐thujaplicin synthesis may involve different genes or it may be synthesized earlier in a shared pathway when compared to the other wood thujaplicins. This is consistent with the large number of SNPs unique to β‐thujaplicin among wood thujaplicins (1634), and the relatively low genetic correlation with γ‐thujaplicin and β‐thujaplicinol (~0.2).

Height and DBH, expectedly, had by far the largest number of shared SNPs (1619) – likely in large part due to their strong genetic correlation (0.75) and the larger number of SNPs overall associated with these traits (Figure 2d). Of these SNPs, 1086 were in 662 genes, indicating that almost a third of SNPs associated with both of these traits are in intergenic regions of the genome. This pattern is similar for all SNPs associated with each of these growth traits.

#### 3.5.2 Localization of trait‐associated SNPs

Using a putative linkage map for WRC established by Shalev et al. (2022), we mapped SNPs for each trait onto their respective LGs, to determine how these SNPs are distributed across the genome, and where SNPs of greatest ES are located. LG 2 had the largest proportion of SNPs (14.8%), closely followed by LG 1 and LG 4 with 12.9% and 10.8% of all SNPs each, respectively. Standardized to the size of each putative giant sequoia chromosome, LG 2 still had the highest proportion of all SNPs (Table S5).

Large‐effect SNPs for α‐thujone were distributed across all putative LGs, with more than 20% on LG 2, although this was not significantly more than would have been expected given the distribution of all SNPs (*p* = 0.0614, Fisher's exact test) (Figure S4). This includes one SNP with an estimated ES nearly seven times larger than any other, which is located in a gene putatively annotated as encoding for a ubiquitin‐associated domain (UBA/TN‐S). The same pattern and significance level were observed for β‐thujone. Sabinene, on the other hand, had over 30% of its large‐effect SNPs on LG 10, which is significantly more than expected (*p* = 1.35 × 10^−4^). For β‐thujone and sabinene, the SNPs with the largest ES were in intergenic regions on LG 8 and 10 respectively.

Large‐effect SNPs for α‐thujaplicin were significantly over‐represented on LG 2 (*p* = 0.0202), while large‐effect SNPs for γ‐thujaplicin and β‐thujaplicinol were significantly over‐represented on LG 9 (*p* < 8 × 10^−4^). Interestingly, the SNPs with the largest ES for α‐ and β‐thujaplicin were both on LG 9, while for γ‐thujaplicin and β‐thujaplicinol, they were on LG 10 and LG 6, respectively. The largest‐effect SNPs for α‐thujaplicin and β‐thujaplicin were on LG 9, downstream of a gene encoding for a plant protein of unknown function, and in a gene putatively encoding for an ABC family transporter respectively. The largest‐effect SNPs for γ‐thujaplicin and β‐thujaplicinol were in intergenic regions on LG 10 and 6 respectively.

Effect size for height and DBH SNPs was generally small, even for large‐effect SNPs. This may be expected due to the polygenic nature of these traits. The SNPs with the largest ES for height and DBH were found on LG 3 and 8, respectively, and were both in intergenic regions.

### 3.6 Inbreeding has little impact on growth and chemistry traits even during extreme selfing

Western redcedar is known to resist inbreeding depression, and readily self‐fertilizes to produce viable offspring (Russell et al., 2003; Russell & Ferguson, 2008; Shalev et al., 2022; Wang & Russell, 2006). Using our TP, we aimed to test whether inbreeding depression was significant for WRC growth and terpene chemistry traits of interest.

The trees in our TP had a wide range of estimates for the inbreeding coefficient *F*, with a mean of 0.23 and some as high as 0.85 (Figure S5). This was somewhat expected, given WRC's low genetic diversity, and the known presence of self‐pollinated and closely related individuals in the TP (Gamal El‐Dien et al., 2022; Shalev et al., 2022). We found no significant effect of *F* on any of the tested traits, with only height and DBH showing an effect larger than the standard error. A similar trend was observed in our SLs, with no significant effect of *F* on height or foliar monoterpene BVs (Table 2).

**TABLE 2:** Estimates of inbreeding depression derived from mixed linear models for training population data (A) and OLS for selfing lines (B)

(A)			
Trait	Inbreeding depression (SE)	*F*‐statistic (Wald's test)	*p*‐value
Height (cm)	−41.1 (21.8)	3.50	0.0599
DBH (mm)	−8.61 (5.29)	2.60	0.104
Total foliar monoterpenes (μg/g DW)	373.8 (2389.6)	0.00	0.876
Total wood thujaplicins (μg/g CW)	−106.9 (138.0)	0.60	0.439
Sapwood (mm)	0.349 (1.22)	0.10	0.775
Heartwood (mm)	0.959 (1.96)	0.20	0.626
Sapwood‐to‐heartwood ratio	0.0188 (0.0339)	0.30	0.579

(B)			
Trait	Inbreeding depression (SE)	*F*‐statistic	*p*‐value
Height breeding values (cm)	−0.0127 (0.813)	0.00020	0.988
Total foliar monoterpene breeding values (μg/g DW)	−395.0 (1818.0)	0.0472	0.828

#### 3.6.1 Selection for growth remains viable during selfing despite large inbreeding coefficients

**FIGURE 3:**
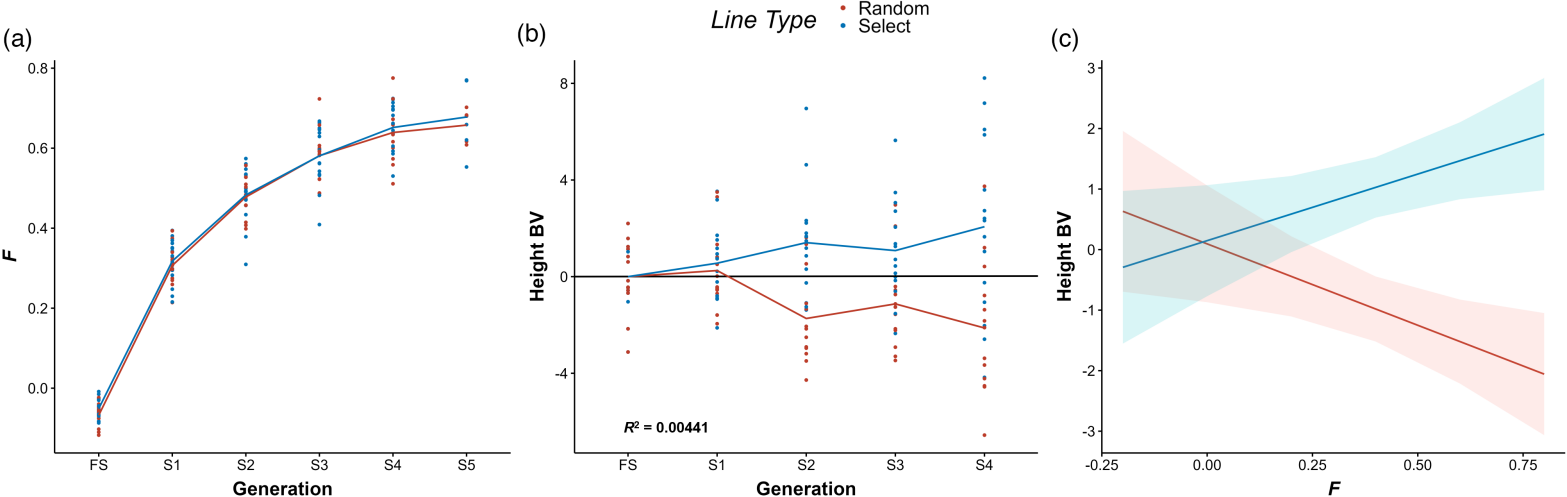
Despite a noticeable increase in the inbreeding coefficient *F* in all selfing line trees with each successive selfing generation, the only significant predictor of height growth was whether a line was under deliberate artificial selection for height. (a) Change in *F* during selfing. *F* increases with each selfing generation similarly within both random and select lines. (b) Change in height BV during selfing. Height BVs increased with each selfing generation in select lines, while height BVs in random lines declined somewhat. Black line represents the regression of height BV on generation, indicating that change in height is not significant when line type is ignored. (c) Interaction plot of predicted height BV and *F*. In the absence of selection, increasing values of *F* are negatively correlated with growth. However, under selection, height BV increases despite an increase in *F*, indicating the effects of inbreeding are readily mitigated by moderate‐selective pressures in WRC.

Given the lack of apparent inbreeding depression even during selfing, we explored the effect of selection for height growth during selfing. Overall *F* increased similarly in both select and random lines (Figure 3a). However, overall height increased each generation in the select lines, while decreasing in the random lines (Figure 3b). There was a significant interaction of *F* and line type, with height declining with increasing *F* in random lines, but increasing in select lines (Figure 3c). Thus, the only significant predictor of height growth during complete selfing was whether a line was select or random, indicating that despite a stark increase in *F*, selection for height growth is viable in WRC (Table S6).

## 4 DISCUSSION

Understanding the genetic basis of traits is fundamental to a wide array of applications, from crop and animal breeding to targeted medicine (Isik et al., 2015; Timpson et al., 2018). Here, we examine the breeding population of an ecologically and economically important conifer species, WRC, and shed light on the genetic architecture of important chemical traits involved in pest and pathogen resistance as well as key growth traits. In addition, using our breeding population and an array of selfing lines, we demonstrate that WRC does not display inbreeding depression for any of these traits.

### 4.1 Genetic architecture varies even among similar traits

Our approach to understanding the genetic architecture of specialized terpene chemistry and growth traits in WRC involved a Bayesian model framework as implemented in BayesR (Moser et al., 2015). Genome‐wide association studies (GWAS), where individual SNPs are regressed against the phenotype, are commonly performed to assess significant loci for traits in large populations. However, due to the large number of SNPs being assessed and the potentially polygenic nature of many complex traits, these studies run the risk of being perpetually underpowered to give a significant *p*‐value to SNPs that in fact do have some effect (Boyle et al., 2017; Yang et al., 2010). The approach of BayesR sidesteps this limitation by fitting all SNPs simultaneously to the model and assigning them to different effect sizes based on their posterior probability of tracking quantitative trait loci associated with the trait of interest (Goddard et al., 2016). Indeed, many complex traits have been found to be more complex than once thought, especially growth traits, which appear to have a large polygenic component (Boyle et al., 2017; Watanabe et al., 2019; Wray et al., 2018; Yang et al., 2015). Less is known about the genetic architecture of specialized chemistry traits, although many of the biochemical pathways for the synthesis of metabolites such as terpenoids have been partially or fully elucidated, suggesting a certain measure of major gene control (Keeling & Bohlmann, 2006; Nützmann & Osbourn, 2014; Tholl, 2015). Terpene synthases (TPS), in particular, have been especially well characterized (Booth et al., 2020; Chen et al., 2011; Shalev et al., 2018).

Thus, we initially expected terpene traits to be mostly impacted by a few major loci of large effect, while growth traits would be mostly polygenic. Although this was largely true of the growth traits surveyed here, there was some variability among terpene traits. In particular, sabinene had the highest proportion of polygenic‐effect SNPs of any trait examined, and all terpene traits had between 30% and 50% of their variance explained by small‐ or polygenic‐effect SNPs. This suggests that, despite the presence of major genes controlling which metabolite is synthesized, these traits are complex and phenotypic variation can be polygenic. We also observed some clustering of large‐effect SNPs based on our putative linkage map of the WRC genome (Shalev et al., 2022). Although SNPs with some effects were distributed across all LGs for each trait, certain LGs had greater proportions of large‐effect SNPs; for example, LG 2 for α‐ and β‐thujone and α‐thujaplicin, LG 9 for γ‐thujaplicin and β‐thujaplicinol and LG 8 for sabinene. Overall, the more polygenic a trait was, the more evenly distributed large‐effect SNPs were across all LGs, whereas major‐effect loci were more likely to be localized to specific LGs. This may suggest linked groups of genes or potentially genetic hitchhiking. Shalev et al. (2022) report that LD decays slowly in WRC. This may increase the probability of genetic hitchhiking, and may also confound observations of SNPs associated with traits. In particular, it may be difficult to fully disentangle here the effects of intergenic SNPs and their association with traits from potentially linked but distant loci that may actually be associated with the trait. Nonetheless, we can take away two important outcomes from our results: first, there are typically loci with a much larger‐effect size than others for each trait that will be important for assessing and further studying; and second, certain LGs are more likely to contain loci associated with certain traits, meaning that a focus on these LGs will be helpful when addressing a specific trait. Future improvement of the genome assembly and annotation will further aid in increasing the resolution of our localization of associated loci.

The importance of the terpene traits examined in this study to defence against deer browse and heartwood rot, and thus to WRC breeding, has been previously described (Morris & Stirling, 2012; Russell & Yanchuk, 2012; Vourc'h, Russell, et al., 2002); given the observed differences in genetic and phenotypic correlations for these traits, fully developing the biosynthetic pathways of these terpenes will be fundamental to understanding the mechanisms controlling their expression. The utility of the trait‐associated SNPs identified in this study is twofold: first, they will provide important gene targets for functional characterization of the biosynthetic pathways of terpene chemistry traits in WRC; and second, they will allow for development of a targeted SNP‐genotyping panel for growth and terpene traits in operational breeding programmes.

### 4.2 WRC is unaffected by inbreeding depression for chemistry and growth traits

One of the most unique aspects of WRC biology is a mixed‐mating system characterized by relatively low outcrossing rates in nature (~70%; El‐Kassaby et al., 1994; O'Connell et al., 2001, 2004). This is contrary to observations in other conifers, such as pines (Remington & O'Malley, 2000; Sorensen & Miles, 1974; Williams, 2008), firs (Sorensen, 1982, 1999) and douglas fir (Sorensen, 1999; Sorensen & Miles, 1974), which all display high levels of outcrossing and strong inbreeding depression. Molecular evidence suggests that during the most recent glacial period, WRC were isolated to a single refugium near the southern end of its current range (O'Connell et al., 2008; Shalev et al., 2022). Given the pattern and rate of migration and continuous distribution of WRC, it is likely that its successful dispersal since the end of the last glaciation is related to its unique mating system, allowing for colonization of new areas while isolated from other conspecifics (Baker, 1955; Pannell & Barrett, 1998).

Previous studies have found inbreeding depression for seed traits to be practically nonexistent, while the effect on growth and productivity appears to be low (Russell et al., 2003; Wang & Russell, 2006). Using a pedigreed, genotyped and extensively phenotyped TP for genomic selection, we were able to model and assess whether inbreeding depression is significant in a real‐world scenario for WRC breeding. Our estimates of *F* from genotyping ranged widely, with some individuals having estimates as high as 0.85. Despite a high mean *F* (0.23), inbreeding depression was not significant for any of the growth or chemistry traits we tested. Russell and Ferguson (2008) found previously that selection for height growth is possible even under complete selfing. Using genotyping data, we show that this response to selection occurs despite sharp increases in *F* with each selfing generation and that even in random lines, inbreeding depression is not a significant predictor of height. Selfing rates tend to be higher in seed orchards (up to 60%; El‐Kassaby et al., 1994; Glaubitz et al., 2000) than in natural populations, and there has been some debate over the merit of attempting to reduce these rates during breeding for operational forestry (Ritland et al., 2020; Russell et al., 2003; Wang & Russell, 2006). Wang and Russell (2006) estimate that 8.1% of merchantable volume on average will be lost by age 60 due to selfing; however, they also suggest that this effect may be mitigated by planting site improvement and sufficient selection intensity. Recently, Gamal El‐Dien et al. (2022) were able to use the same TP from this study to successfully generate genomic selection predictions which capture LD for implementation of genomic selection in WRC, which is ongoing. We add further that even individuals with high *F* estimates can be effective targets for selection and breeding, underscoring the importance of understanding the interactions of causal loci influencing the expression of key traits and increasing selection intensity for these loci, rather than attempting to drastically reduce inbreeding in WRC breeding populations.

## 5 CONCLUSIONS

The aim of this study was to identify the genomic regions which may be associated with ecologically and economically important terpene chemistry and growth traits in WRC, and to explore whether WRC's tendency towards inbreeding might suggest a lack of inbreeding depression for such traits. We found evidence of complex polygenic genetic control for all terpene and growth traits surveyed, with most terpene traits potentially retaining some level of major gene control on specific LGs in the genome. Moreover, we found no evidence of inbreeding depression for either terpene or growth traits in WRC. In addition to providing an important resource for WRC management and improvement, our results showcase the importance of assessing the polygenicity and interconnected nature of traits and the loci associated with them in models of genetic architecture.

## CONFLICT OF INTEREST

The authors declare no conflict of interest.

## Supporting information


Appendix S1

Dataset S1

Dataset S2

Dataset S3

Dataset S4

## Data Availability

The SNP data generated in this study have been submitted to the Zenodo data repository under DOI 10.5281/zenodo.6562381. Code and raw data used for generating data files and figures and Supplemental Dataset files have been uploaded to the following GitHub repository: https://github.com/tshalev/WRC‐traits‐inbreeding‐paper. Supplemental Dataset files and Supplemental Code are available as Supplemental Information.
