# Supplementary material for: Genetic architecture of terpene chemistry and growth traits and the impact of inbreeding on these traits in western redcedar (Thuja plicata)
Source: Evol Appl. 2023 Jan 12;16(3):673–87. doi: 10.1111/eva.13526 (PMC10033848; doi:10.1111/eva.13526)
Supplement: Supplementary file 1 — Appendix S1 [file EVA-16-673-s002.pdf]

Supplemental Information for

Genetic architecture of terpene chemistry and growth traits and the impact of inbreeding on these traits in western redcedar (*Thuja plicata*)

Tal J. Shalev, Omnia Gamal El-Dien, Macaire M. S. Yuen, Lise van der Merwe, Matias Kirst, Alvin D. Yanchuk, Carol Ritland, John H. Russell, Joerg Bohlmann

*Corresponding authors: Tal J. Shalev and Joerg Bohlmann.
Email: tal.shalev@msl.ubc.ca; bohlmann@msl.ubc.ca

This PDF file includes:

Figures S1 to S5

Tables S1 to S7

Summaries for Datasets S1 to S5

Summaries for Code S1 to S8

SI References

Other supplementary materials for this manuscript include the following:

Datasets S1 to S5

Code S1 to S8

FIGURES

A

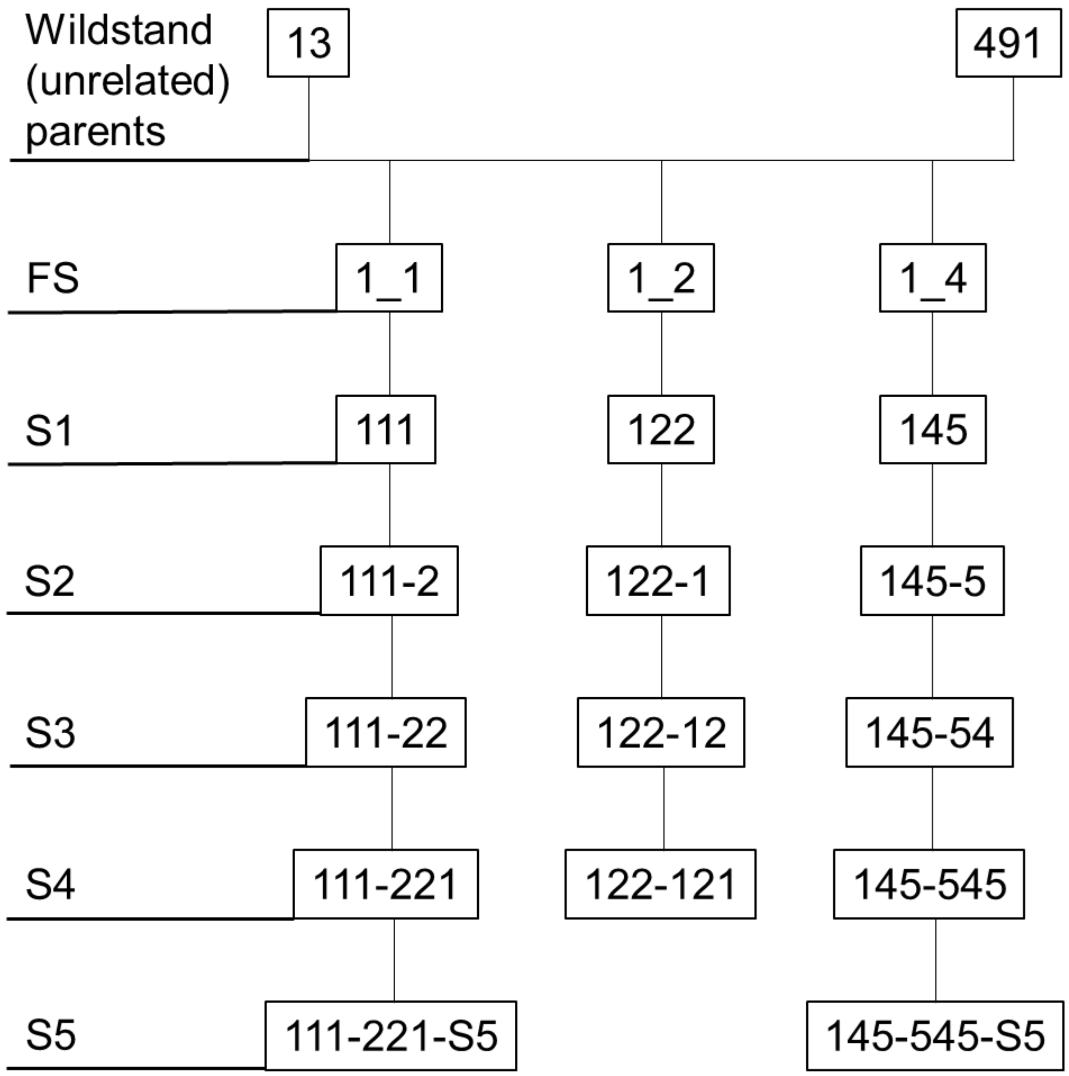

B

Selfing Line	FS	S1	S2	S3	S4	S5
1	1_1	111	111-2	111-22	111-221	111-221-S5
1	1_2	122	122-1	122-12	122-121	
1	1_4	145	145-5	145-54	145-545	145-545-S5
6	6_1	611	611-1	611-11	611-111	
6	6_2	623	623-2	623-21	623-211	
6	6_4	646	646-4	646-45	646-454	646-454-S5
7	7_1	712	712-1	712-11	712-111	
7	7_2	721	721-2	721-21	721-211	
7	7_4	745	745-4	745-44	745-444	
8	8_2	821	821-1	821-11	821-111	
8	8_4	845	845-4	845-44	845-444	
8	8_5	854	854-5	854-54	854-544	
10	10_1	1011	1011-1	1011-12	1011-121	
10	10_2	1022	1022-1	1022-11	1022-113	1022-113-S5
10	10_5	1056	1056-4	1056-45	1056-454	
13	13_2	1323	1323-2	1323-21	1323-211	
13	13_3	1332	1332-1	1332-11	1332-111	
13	13_4	1345	1345-5	1345-54	1345-544	
16	16_1	1611	1611-2	1611-21	1611-211	
16	16_2	1621	1621-2	1621-22	1621-221	
16	16_5	1654	1654-4	1654-45	1654-454	1654-454-S5
17	17_2	1721	1721-1	1721-11	1721-111	1721-111-S5
17	17_4	1744	1744-5	1744-54	1744-544	
17	17_5	1755	1755-5	1755-54	1755-545	1755-545-S5
19	19_1	1912	1912-1	1912-13	1912-131	
19	19_2	1922	1922-1	1922-11	1922-111	
19	19_5	1955	1955-5	1955-54	1955-544	1955-544-S5
20	20_1	2013	2013-1	2013-13	2013-131	
20	20_4	2045	2045-5	2045-54	2045-544	
21	21_1	2111	2111-1	2111-11	2111-111	
21	21_2	2121	2121-1	2121-11	2121-111	2121-111-S5
21	21_6	2165	2165-4	2165-44	2165-444	2165-444-S5
23	23_2	2323	2323-2	2323-21	2323-211	2323-211-S5
23	23_4	2344	2344-4	2344-46	2344-464	
26	26_1	2612	2612-1	2612-12	2612-121	
26	26_4	2645	2645-4	2645-46	2645-464	
27	27_1	2712	2712-2	2712-21	2712-211	
27	27_2	2721	2721-1	2721-12	2721-121	
27	27_6	2765	2765-5	2765-55	2765-554	
29	29_2	2923	2923-1	2923-12	2923-121	2923-121-S5
29	29_4	2944	2944-5	2944-56	2944-565	2944-565-S5

Figure S1: Schematic of breeding and genotyping strategy employed for the western redcedar selfing lines (SLs). A) Sample family tree for one cross of unrelated parent trees, resulting in three separate, complete selfing lines. The first digit in each sample ID is the line identifier. Each subsequent digit identifies the seedling that was chose to continue that SL. Every generation adds an additional digit to assign a unique identifier to each individual up until the S4 generation. S5 is indicated by adding S5 to the end of the identifier. Lines in which the second digit is 1, 2, or 3 (e.g., 1_1) were selected for height breeding values. Lines in which the second digit is 4, 5, or 6 were randomly chosen. **B)** Summary of all SLs used in the study, adapted from Shalev et al. (2022). Green: Sample genotyped and correctly labeled; Red: Sample genotyped and incorrectly labeled; Blue: Sample genotype manually imputed; Grey: Sample not sequenced (dead/missing); White: Sample not sequenced.

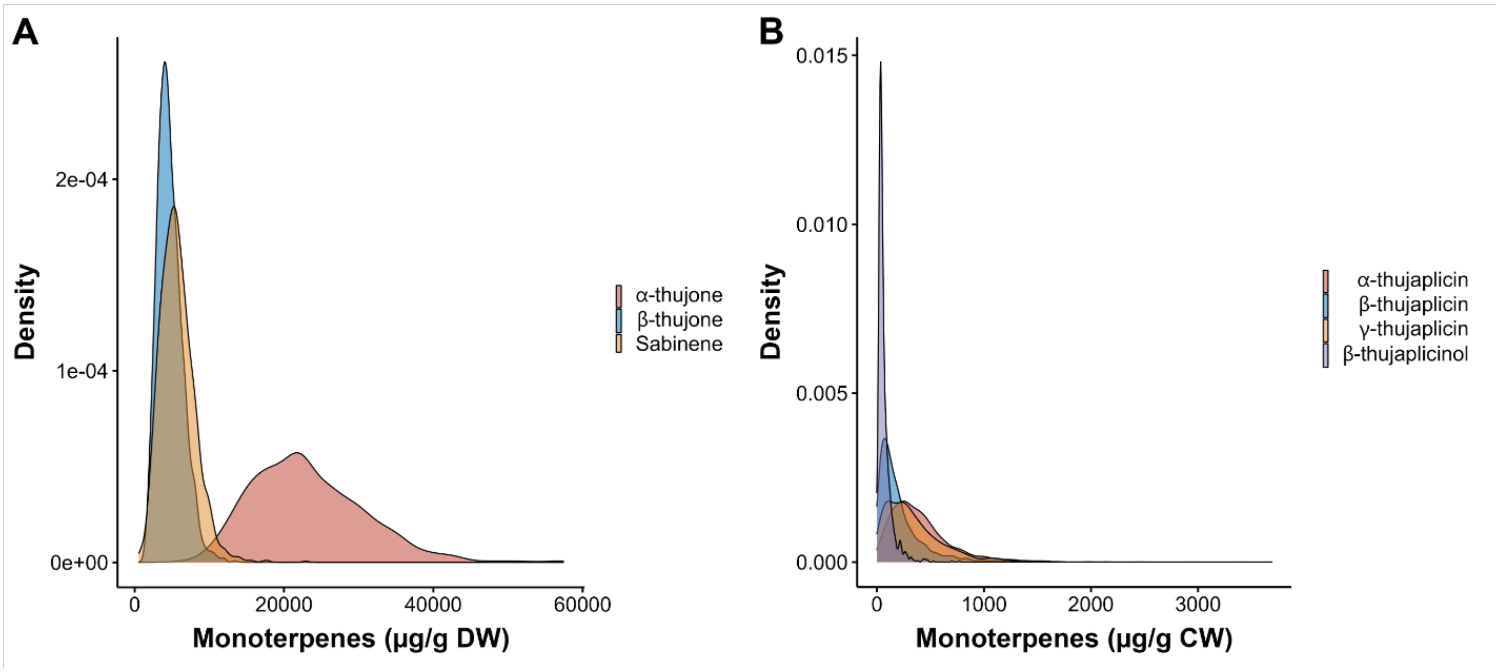

Figure S2: Density plots of the amounts of different foliar (A) and wood (B) monoterpenes in the training population. Density indicates the proportion of observations of a range of terpene concentrations.

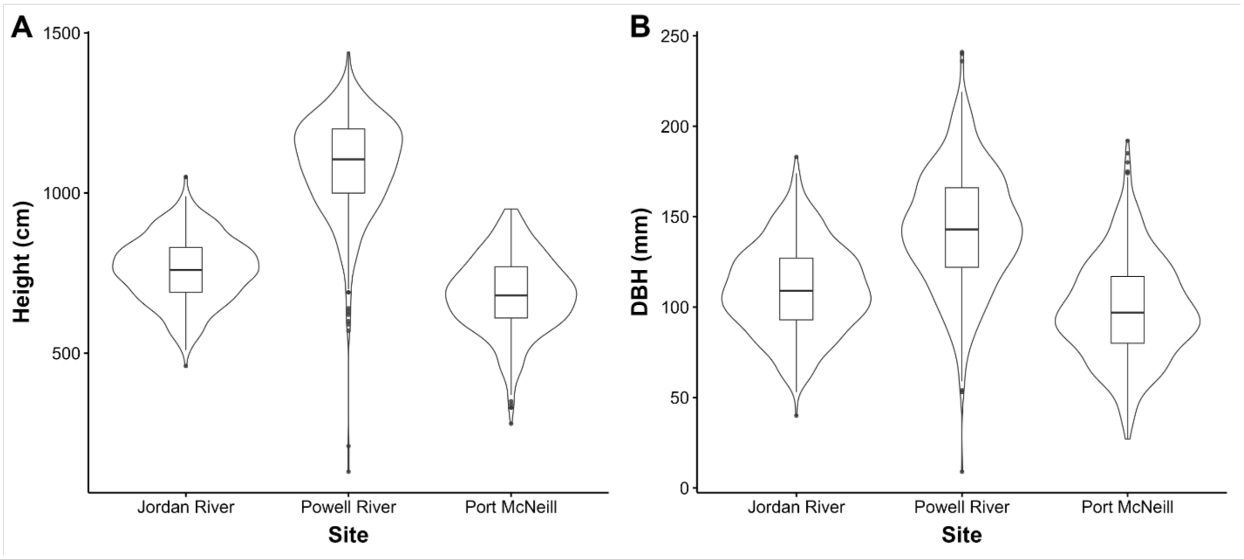

Figure S3: Distribution of height (A) and diameter at breast height (DBH; B) across the three progeny test sites in the training population.

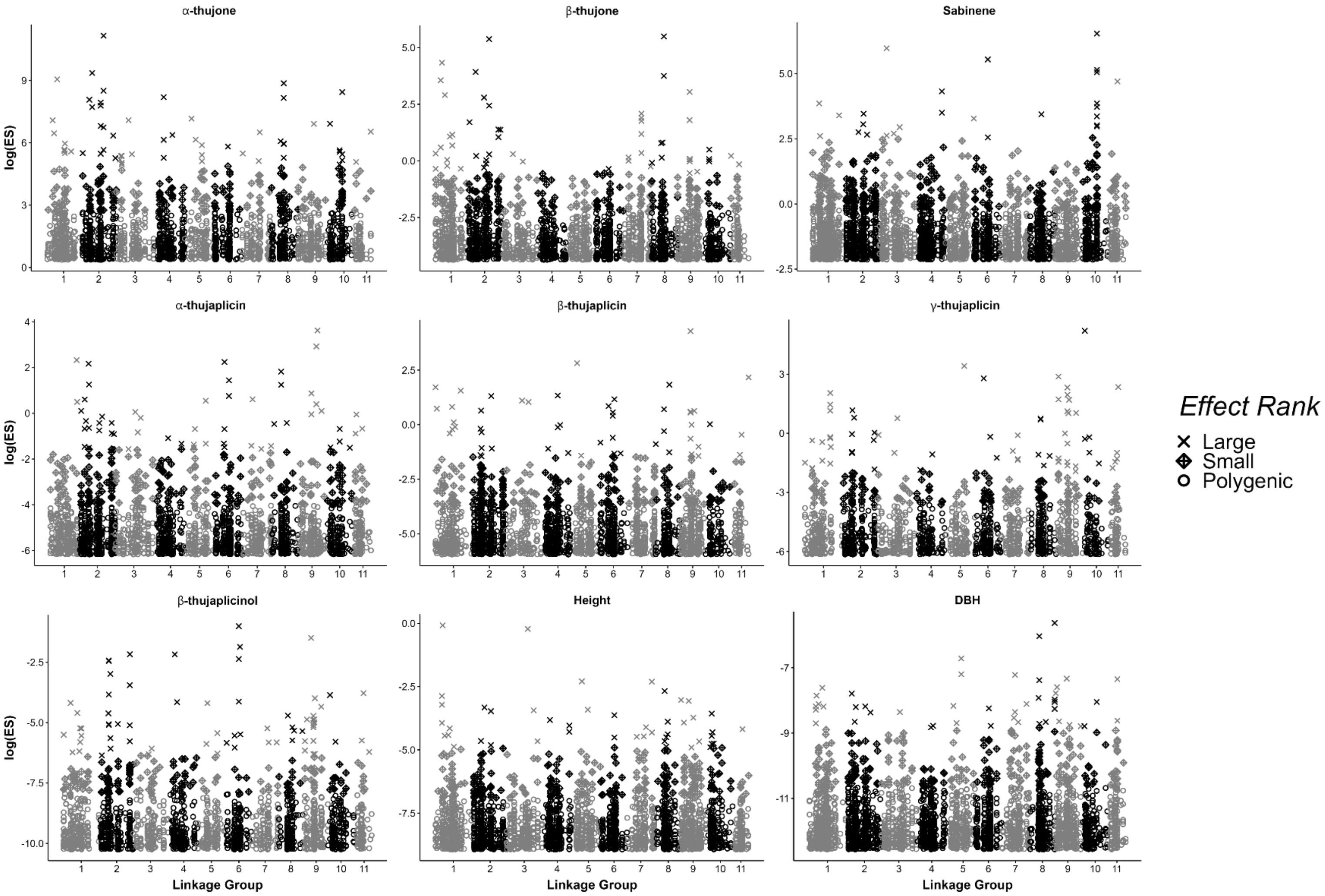

Figure S4: Distribution of SNPs of three putative effect sizes as determined using BayesR (Moser et al., 2015)**, mapped across putative linkage groups based on the draft genome of *Sequoiadendron giganteum* (giant sequoia)** (Scott et al., 2020)**.**

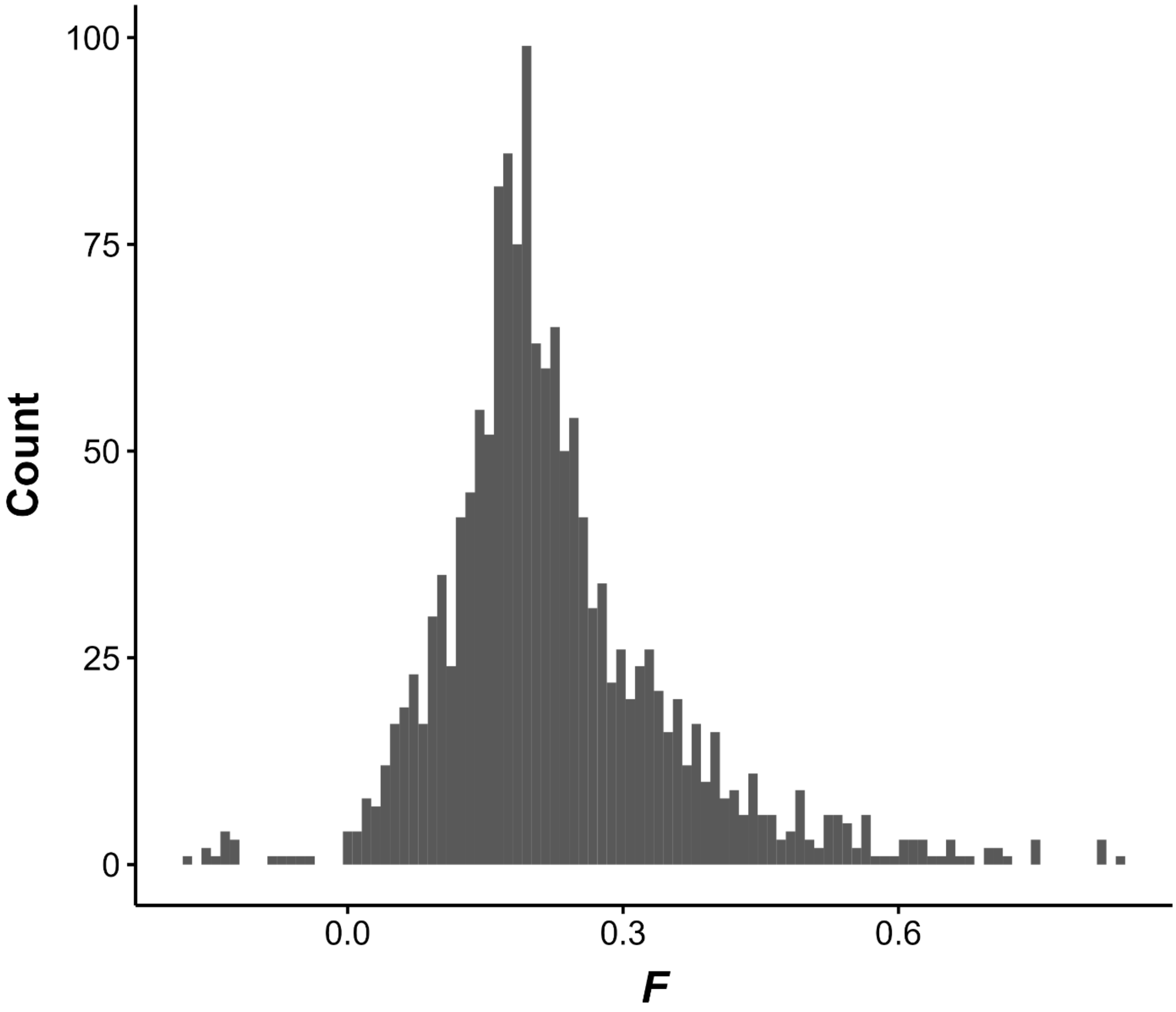

Figure S5: Distribution of the inbreeding coefficient *F* as determined by the approach of (Yang et al. 2011) **in the training population.**

TABLES

Table S1: Models used to predict breeding values (BVs) and estimate adjusted phenotypes for the genomic selection training population. For prediction of BVs, the inverse genomic realized relationship matrix (GRRM) was included as a random effect in the model. Model terms preceded with 'idh' used a block diagonal (heterogeneous) variance structure to split the variance for that term.

Trait category	Trait	Fixed effects	Random effects
**Growth**	HT15 *	site	idh(site)×replicate + idh(site)×replicate×block
	DBH15 *	site	site×replicate + site×replicate×block
**Foliar terpenes**	α-thujone	site	idh(site)×replicate + idh(site)×replicate×block
	β-thujone	site	site×replicate + idh(site)×replicate×block
	Sabinene	site	site×replicate + site×replicate×block
	Total foliar monoterpenes ǂ	site	idh(site)×replicate + site×replicate×block
**Wood terpenes**	α-thujaplicin	site + pith age + SHR	site×replicate + site×replicate×block
	β-thujaplicin	site	-
	γ-thujaplicin	site + pith age	site×replicate + site×replicate×block +
	β-thujaplicinol	site + pith age + SHR	-
	Total wood thujaplicins ǂ	site + DBH15 + pith age	idh(site)×replicate + site×replicate×block
**Dendrochronology**	Sapwood ǂ	site + DBH15 + pith age	idh(site)×replicate + idh(site)×replicate×block
	Heartwood ǂ	site + DBH15 + pith age	idh(site)×replicate + idh(site)×replicate×block
	Sapwood to heartwood ratio ǂ	site + DBH15 + pith age	idh(site)×replicate + idh(site)×replicate×block

HT15 – Height at 15 years

DBH15 – Diameter at breast height at 15 years

CLB15 – Cedar leaf blight incidence at 15 years

SHR – Sapwood to heartwood ratio

* – Also used to estimate inbreeding depression, with inbreeding coefficient F added as a fixed effect

ǂ – Only used for estimation of inbreeding depression

Table S2: Student's *t*-test for differences in height and DBH for the three different test sites in the training population. Two-sample one-tailed *t*-tests were performed to assess whether height and DBH were significantly greater at the Powell River site when compared to the Jordan River and Port McNeill sites.

**Site**	**Average height (cm) (SD)**	**Site Comparison**	***t***	***p*-value**
(1) Jordan River	761.75 (102.12)	2/1	39.44	$< 2\times10^{-16}$
(2) Powell River	1084.85 (156.43)	2/3	45.84	$< 2\times10^{-16}$
(3) Port McNeill	683.39 (119.25)	-	-	-
**Site**	**Average DBH (mm) (SD)**	**Site Comparison**	***t***	***p*-value**
(1) Jordan River	109.93 (24.44)	2/1	18.51	$< 2\times10^{-16}$
(2) Powell River	143.07 (32.72)	2/3	23.16	$< 2\times10^{-16}$
(3) Port McNeill	98.67 (27.87)	-	-	-

Table S3: Narrow-sense heritability (h^2) estimates from genomic realized relationships (G) and pedigree (A).

Trait	G h^2 (SE)	A h^2 (SE)
**α-thujone**	0.27 (0.047)	0.35 (0.075)
**β-thujone**	0.16 (0.041)	0.14 (0.043)
**Sabinene**	0.27 (0.045)	0.39 (0.078)
**α-thujaplicin**	0.26 (0.045)	0.26 (0.061)
**β-thujaplicin**	0.30 (0.044)	0.21 (0.049)
**γ-thujaplicin**	0.19 (0.038)	0.20 (0.051)
**β-thujaplicinol**	0.13 (0.032)	0.11 (0.034)
**Height**	0.14 (0.034)	0.15 (0.044)
**DBH**	0.10 (0.032)	0.10 (0.036)

Table S4: Genetic (lower triangle) and phenotypic (upper triangle) correlations between all study traits estimated using genomic realised relationships (A) and pedigree (B).

(A)

	Height	DBH	α-thujone	β-thujone	Sabinene	α-thujaplicin	β-thujaplicin	γ-thujaplicin	β-thujaplicinol
Height	-	0.85 ++	0.055 +	-0.043 +	-0.011	-0.032 +	-0.11 ++	-0.011	-0.12 ++
DBH	0.75 ++	-	0.098 ++	-0.036 +	0.033 +	-0.10 ++	-0.15 ++	-0.067 ++	-0.18 ++
α-thujone	0.031	0.19 +	-	0.60 ++	0.66 ++	-0.027	-0.020	-0.033 +	-0.061 ++
β-thujone	0.20 +	0.39 +	0.65 ++	-	0.32 ++	-0.035 +	0.019	-0.0050	-0.026 +
Sabinene	-0.076	-0.30	0.35 ++	0.30 ++	-	0.022	-0.0097	-0.023	-0.027
α-thujaplicin	0.082	-0.051	0.11	0.12	0.31 ++	-	0.54 ++	0.54 ++	0.47 ++
β-thujaplicin	0.027	0.12	0.17 +	0.10	0.058	0.46 ++	-	0.41 ++	0.36 ++
γ-thujaplicin	0.030	0.028	0.071	-0.023	0.074	0.53 ++	0.22 +	-	0.76 ++
β-thujaplicinol	-0.10	-0.069	-0.20 +	-0.20 +	-0.0090	0.35 ++	0.20 +	0.76 ++	-

(B)

	Height	DBH	α-thujone	β-thujone	Sabinene	α-thujaplicin	β-thujaplicin	γ-thujaplicin	β-thujaplicinol
Height	-	0.84 ++	0.056 +	-0.031 +	-0.011	-0.025	-0.10 ++	-0.014	-0.13
DBH	0.72 ++	-	0.11 ++	-0.015	0.031	-0.10 ++	-0.14 ++	-0.060 ++	-0.18 ++
α-thujone	0.082	0.29 +	-	0.61 ++	0.63 ++	-0.018	-0.0056	-0.042 +	-0.076 ++
β-thujone	0.28 +	0.43 +	0.73 ++	-	0.32 ++	-0.031 +	0.026	-0.015	-0.029 +
Sabinene	0.042	0.032	0.43 ++	0.36 ++	-	0.071 ++	-0.0075	-0.014	-0.028
α-thujaplicin	0.084	-0.18	0.074	0.038	0.59 ++	-	0.53 ++	0.54 ++	0.46 ++
β-thujaplicin	-0.021	0.097	0.33 +	0.19	0.16	0.36 ++	-	0.40 ++	0.34 ++
γ-thujaplicin	-0.074	0.084	-0.16	-0.23 +	0.12	0.56 ++	0.25 +	-	0.75 ++
β-thujaplicinol	-0.25 +	-0.32 +	-0.45 ++	-0.23 +	-0.060	0.21 +	0.045	0.67 ++	-

+: Estimate larger than the standard error

++: Estimate more than twice as large as the standard error

Table S5: Number of SNPs in each putative linkage group derived from the *Sequoiadendron giganteum* (giant sequoia) draft genome (Scott et al., 2020)**.** Percentage of total was scaled by the length of each putative giant sequoia chromosome.

Linkage group	# SNPs	% Total	Scaled % total
**1**	4475	12.9	9.40
**2**	5149	14.8	12.2
**3**	3023	8.69	7.43
**4**	3741	10.7	10.7
**5**	2512	7.22	7.54
**6**	3014	8.67	9.23
**7**	2907	8.36	9.14
**8**	2875	8.27	9.17
**9**	3401	9.78	11.0
**10**	2178	6.26	7.14
**11**	1493	4.29	6.98

Table S6: Effect of inbreeding on growth during selfing. In four generations of complete selfing, deliberate selection for seedling height was the only significant predictor of height, despite an increase in the inbreeding coefficient *F* with each selfing generation (one-way ANOVA; OLS linear model). Moreover, there was a significant interaction effect of *F* and type, as predicted height BVs of random lines declined while those of select lines increased (Figure 3C).

**Variable**	**Effect (SE)**	**F-statistic**	***P*-value**
**Type (Select/Random)**	0.0532 (0.679)	27.8	5.18×10^{-7}
F	-2.69 (1.06)	0.0003	0.987
**Type:*F* (interaction)**	4.89 (1.45)	11.4	9.42×10^{-4}

DATASETS

Dataset S1: Master phenotypic dataset containing all study design, foliar chemistry, wood chemistry, growth, dendrochronology, and inbreeding data for the training population in this study.

Dataset S2: Adjusted phenotypes for all assessed foliar terpene, wood terpene, and growth traits.

Dataset S3: Inbreeding, height BV, foliar terpene, and sample collection data for the selfing lines in this study.

Dataset S4: Final filtered SNP dataset (n = 36, 262) for the training population.

Dataset S5: Putative trait-associated SNPs for all assessed traits in the study.

CODE

Code S1: Phenotypic distribution plots.

Code S2: Bivariate models (pedigree).

Code S3: Bivariate models (genomic relationships).

Code S4: BayesR SNP mixture distributions, plot for Figure 1.

Code S5: SNP effect sizes, mapping SNPs to linkage groups.

Code S6: SNP annotation.

Code S7: UpSet plots for trait overlap, overlapping SNPs, plot for Figure 2.

Code S8: Inbreeding depression analysis, plot for Figure 3.

REFERENCES

Moser, G., Lee, S. H., Hayes, B. J., Goddard, M. E., Wray, N. R., & Visscher, P. M. (2015). Simultaneous Discovery, Estimation and Prediction Analysis of Complex Traits Using a Bayesian Mixture Model. *PLoS Genetics*. https://doi.org/10.1371/journal.pgen.1004969

Scott, A. D., Zimin, A. V., Puiu, D., Workman, R., Britton, M., Zaman, S., Caballero, M., Read, A. C., Bogdanove, A. J., Burns, E., Wegrzyn, J., Timp, W., Salzberg, S. L., & Neale, D. B. (2020). A reference genome sequence for giant sequoia. *G3: Genes, Genomes, Genetics*, 10(11), 3907–3919. https://doi.org/10.1534/g3.120.401612

Shalev, T. J., Gamal El-Dien, O., Yuen, M. M. S., Shengqiang, S., Jackman, S. D., Warren, R. L., Coombe, L., van der Merwe, L., Stewart, A., Boston, L. B., Plott, C., Jenkins, J., He, G., Yan, J., Yan, M., Guo, J., Breinholt, J. W., Neves, L. G., Grimwood, J., … Bohlmann, J. (2022). The western redcedar genome reveals low genetic diversity in a self-compatible conifer. *Genome Research*, 32(10). https://doi.org/10.1101/GR.276358.121

Yang, J., Lee, S. H., Goddard, M. E., & Visscher, P. M. (2011). GCTA: A tool for genome-wide complex trait analysis. *American Journal of Human Genetics*, 88(1), 76–82. https://doi.org/10.1016/j.ajhg.2010.11.011
